# Single-Channel Flow Injection Spectrophotometric Determination of Nickel Using Furildioxime in Micellar Solution

**DOI:** 10.1100/2012/418047

**Published:** 2012-05-01

**Authors:** Najma Memon, Saima Memon, Amber R. Solangi, Rubina Soomro, Rabel Soomro

**Affiliations:** ^1^NCE in Analytical Chemistry, University of Sindh, Jamshoro 76080, Pakistan; ^2^Institute of Advanced Studies in Chemical Sciences, University of Sindh, Jamshoro 78060, Pakistan

## Abstract

A very simple, selective, and fast flow injection spectrophotometeric method is developed for determination of nickel using furildioxime as complexing agent. Micellar solution of brij-35 is employed to solubilize the sparingly soluble complex of Ni-furildioxime in buffered aqueous system (pH-9.00). Under optimized conditions, absorbance is linear from 0.02 to 10 *μ*g mL^−1^ using 500 *μ*L sample volume and from 10 to 30 *μ*g mL^−1^ using 50 *μ*L sample volume of nickel at 480 nm, with *R* ^2^ = 0.9971 and 0.9916, respectively. The molar absorption coefficient and Sandell's sensitivity were 6.0 × 10^3^ L mol^−1^ cm^−1^ and 0.01 ng cm^−2^, respectively. The sample throughput of the method is 120 samples per hour with RSD of 0.01–0.2% for 0.02 to 10 *μ*g mL^−1^ nickel (*n* = 5), indicating that the method is highly precise and reproducible. Interference from cobalt is removed by Nitroso R-salt-modified XAD-16. The developed method is validated by analysing certified reference materials and is applied to assess nickel content of commercially available cigarettes.

## 1. Introduction

Interest in the determination of nickel has increased over the last few years because of the influence of this metal on humans. Adverse effects of inorganic, water-soluble nickel species occur after contact with skin, causing nickel dermatitis. After inhalation, respiratory tract irritation and asthma can also result. Besides carcinogenensis, bioassays in animals showed that certain nickel compounds are potent carcinogens when administered via parenteral routes [1].

 In rapidly expanding analytical fields, such as environmental, biological, and material monitoring of trace metals, there is an increasing need to develop simple, sensitive, selective, and reproducible analytical techniques that do not use expensive or complicated test equipments. Many sensitive instruments, such as spectrofluorimetry, X-ray fluorescence spectrometry, neutron activation analysis, atomic absorption spectrophotometery, and chemiluminescence have been widely applied for the determination of nickel [2–8]. However, spectrophotometric methods still have the advantages of simplicity and require no expensive or complicated test equipment. Also, there are various automated spectrophotometric methods (flow injection based) for the determination of nickel, but due to extraction of complex in organic solvents, complicated FIA setups are used [9]. A flow Injection visible diffused reflectance method by precipitating nickel with furildioxime was reported but reproducibility of peak heights is not satisfactory [10].

 Spectrophotometrically, nickel is determined by using complexing agents, such as bis(acetylacetone)ethylenediimine, 2-(2-Quinolylazo)-5-diethylaminoaniline, dimethylglyoxime, and furildioxime [9, 11–13].

 Oximes are group of compounds, which reacts with limited number of metal ions, hence come under the umbrella of selective reagents. These form water-insoluble complexes extractable into organic solvents, but the extraction is not 100% due to partial solubility of complexes. Furildioxime forms complex with nickel that is absorbed in chloroform at 438 nm [12]. Inspite of high selectivity of this reagent toward nickel, its use is limited due to sparingly soluble precipitates and solubilizing of Ni-furildioxime [14]. Recently, dissolution of precipitate formed by Ni-furildioxime is tried by ultrasonic irradiation but quick transfer from ultasnoic bath to spectrophotometer is the condition for analysis as turbidity reappears [15].

The ability of surfactants to solubilize the water-insoluble complexes in homogenous environment can overcome this problem. There is only one report that addresses the flow injection determination of nickel using pyridylazoresorcinol in TX-100 solution but it is laboratory exercise published by undergraduate students and proper optimization is not worked out [16]. Micellar solutions in FI have unique feature of sensitivity and speed of analysis with less waste for treatment. Micellar solutions obviate the need of phase separator in FIA systems which resulted in simple setups with more reproducible assays [14, 17, 18].

 This study is aimed at developing simple, rapid, selective, and sensitive assay for nickel. Furildioxime is selected as complexing reagent due to its selective behavior toward nickel. Micellar solutions are used as microheterogonous system to carry out complexation reaction, and flow injection is employed to automate the system and to provide high-throughput determination. Assay is made selective by removing cobalt, major interfering metal ion, by using NitrosoR-salt modified XAD-16.

## 2. Experimental

### 2.1. Apparatus

A four-channel peristaltic pump (Gilson Minipuls 3) was used to propel the carrier stream, equipped with PVC pump tubes with i.d 0.5 mm (anachem). Samples were injected via rotary PTFE valve (Rheodyne 5020). PTFE 0.5 mm i.d. tubing was used throughout the remainder system. The detector used was Spectronic 20 at 488 nm equipped with 100-*μ*L flow through cell. Yew strip chart recorder was used to record the signal. Shimadzu UV-365 spectrophotometer was used to record the spectra of nickel-furildioxime system. A Perkin-Elmer (Lamda-2, Germany) double-beam spectrophotometer was used for the comparison of the results. A digital pH meter (WTW, Inolab pH level 1, Germany) was used to measure the pH of the solution.

### 2.2. Reagents

All chemicals used were of analytical reagent grade or highest purity available. Double-distilled deionized water was used throughout.

### 2.3. Furildioxime Solution

A 5% solution was prepared by dissolving the 5.0 g of furildioxime (Merck, Darmstadt, Germany) in 100 mL of methanol (Merck).

### 2.4. Nickel(II) Standard Solution

A 100 mL stock solution (1 mg mL^−1^) of nickel was prepared by dissolving appropriate amount of nickel sulfate (Fluka, Switzerland) in double-distilled deionized water containing 1 mL of 1 + 1 nitric acid. More diluted solutions were prepared from this stock solution freshly when required in double-distilled deionized water.

### 2.5. Brij-35 Stock Solution (5%)

A 500 mL stock solution of Brij-35 (Merck) was prepared by dissolving 50 g of Brij-35 in 250–300 mL of water and sonicated for 15 minutes and was diluted to mark with double-distilled deionized water when become transparent.

### 2.6. Other Solutions

Solutions of the large number of anions, cations, and complexing agents were prepared from their analaR grade or equivalent grade water-soluble salts. All glassware was kept in nitric acid (1 + 1) for at least a day and then washed with deionized water. Stock solutions and environmental water sample were kept in polypropylene bottles containing 1 mL concentrated nitric acid.

### 2.7. Procedure

#### 2.7.1. Preparation of Reagent for FIA

**Figure 1:**
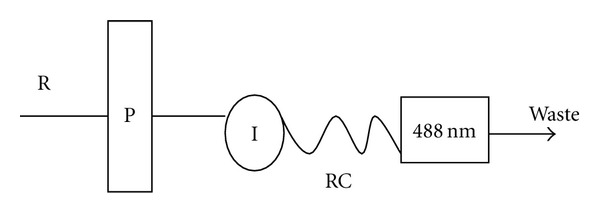
Block diagram for the FIA setup used for determination of nickel using furildioxime.

A single-channel FIA manifold was used throughout. Reagent “R” was prepared by taking 0.05 mL of Brij-35 (5%) and 5.0 mL of buffer (NH_4_Cl/NaOH buffer, pH = 9.00, and *I* = 1) was mixed and 0.4 mL 5% furildioxime was added and mixed well, then diluted to 25.0 mL with deionized water. The reagent was pumped at the flow rate of 2.0 mL min^−1^. The sample was injected at position “*I*” into flowing reagent stream. The sample reacts at point reaction coil “RC” of 100 cm and is detected at 488 nm (Figure 1).

#### 2.7.2. Nitroso-R Salt-Modified XAD-16

Amberlite XAD-16 (5 g) and 50 mL of aqueous 0.1% nitroso-R salt were stirred with the help of magnetic stirrer. Absorbance of nitroso-R salt in solution was monitored continuously using UV-Visible spectrophotometer. When the absorbance of nitroso-R salt became constant, the resin was filtered and dried. The amount of chelating reagent required for the modification of surface of XAD-16 was optimized by varying the concentration and volumes of nitroso-R salt. Recovery (%) of nickel was found to be quantitative using 50 mL of 0.1% nitroso-R salt.

## 3. Results and Discussion

Various chemical and FIA parameters were studied to get optimum signal (absorbance). Studied range, and selected parameters are shown in Table 1.

### 3.1. Factors Affecting Absorbance of Nickel-Furildioxime Complex

#### 3.1.1. Absorption Spectra

The absorption spectra of nickel-furildioxime in Brij-35 at pH 9.00 was recorded using Shimadzu UV-365 spectrophotometer, Japan. The absorption spectrum of nickel-furildioxime shows the maximum absorbance at 480 nm. In all instances, measurements were made at 480 nm against reagent blank.

#### 3.1.2. Effect of Surfactant Type and Concentration

Various types of surfactants; anionic (sodium dodecyl sulfate), cationic (cetyl trimethyl ammonium bromide), and nonionic (Brij-35) were studied; Brij-35 was found to be the best surfactant for the system. Ionic surfactants were rejected for not providing adequate solubility and high background signal which hampers the determination of metal ions at low concentration. In 0.01% Brij-35 medium, however, maximum absorbance was observed; hence 0.01% Brij-35 was used in the determination procedure. It was observed that very little concentration is enough to solublize the complex, while on increasing concentration, the absorbance decreases and remains constant after 0.2% Brij-35. This may be due to micellar dilution effect and also the viscosity of the system increases with surfactant concentration which in turn decreases absorbance [19].

#### 3.1.3. Effect of pH

**Figure 2:**
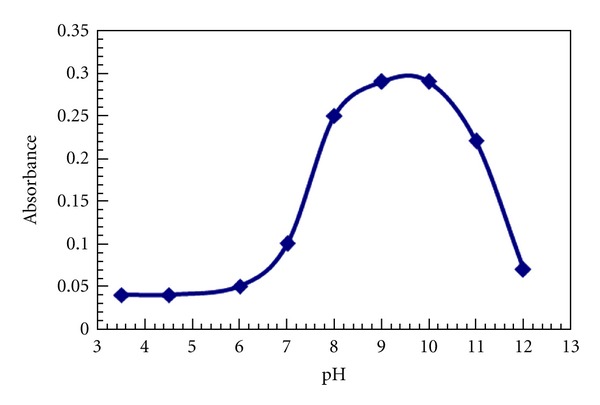
Effect of pH on the absorbance of nickel-fruildiome complex.

Effect of pH was observed by varying pH of reagent from 3.5–12.0 using HCl/NaOH at room temperature. The absorbance was maximum at pH 9.0, using final concentration of buffer 0.2 M at room temperature 25 ± 5°C. Outside this range, the absorbance decreased (Figure 2). This is due to deprotonation of furildioxime at higher pH which favors the formation of complex with positively charged Ni^2+^. The same trend is reported for extraction systems and complexation in aqueous medium, this shows that pH-dependant chemistries are not altered in micellar medium [20]. For all subsequent measurements, 0.2 M buffer (NH_4_Cl/NaOH) at pH 9.0 was used.

#### 3.1.4. Effect of Reagent Concentration

Different volumes of stock 5% methanolic solution of furildioxime were added to prepare reagent “R” for FIA, and fixed concentration of metal ion was injected (50 *μ*L). It was observed that on increasing concentration, signal increases up to 0.1% and then starts decreasing. Low reagent concentration may lead to incomplete complexation, but at higher concentration, the background signal increases and reduces net signal so, reagent concentration of 0.1% was used for further studies.

#### 3.1.5. Effect of FIA Parameters

Flow parameters like pump speed (flow rate), reaction coil length, and sample volume were studied by varying pump speed, home-made reaction coils from Teflon tubes, and loops of different injection volumes, respectively. There is very small effect on the signal on increasing pump speed. Decrease in absorbance on increasing reaction coil length was observed, this shows that the reaction in micellar medium is spontaneous and delaying the reactants in coil will not shift the equilibrium to produce more products. So, reaction coil length of 100 cm and pump speed 12 rpm (corresponds to 2.1 mL min^−1^) was selected. Although, increasing sample volume increased absorbance from 0.2–0.5 for 50–500 **μ**Ls. Large sample volume was used to construct calibration graphs at lower concentrations while 50-*μ*L loop was used at higher concentrations which produce sharp peaks.

#### 3.1.6. Analytical Figures of Merit

The absorbance was linear from 0.02 to 10 *μ*g mL^−1^ using 500 *μ*L sample volume (*y* = 0.0927 + 0.0331) and 10–30 *μ*g mL^−1^ using 50-*μ*L sample volume (**y** = 0.0428 + 0.0764) of nickel at 480 nm, with *R* ^2^ = 0.9971 and 0.9916, respectively. The molar absorption coefficient and Sandell's sensitivity were 6.0 × 10^3^ L mol^−1^ cm^−1^ and 0.01 ng cm^−2^, respectively.

The sample throughput of the method is 120 samples per hour. The RSD (*n* = 5) is 0.01–0.2% for 0.02 to 10 *μ*g mL^−1^ nickel, indicating that the method is highly precise and reproducible. Salient features of proposed method are summarized in Table 2.

#### 3.1.7. Effect of Foreign Ions

The effect of over 40 cations, anions, and complexing agents were studied on the determination of only 1 *μ*g mL^−1^ of nickel. The criterion for interference was an absorbance value varying for more than 5% for the expected value of nickel alone [21]. No interference was found from the following; 1000-fold amount of sulfate, sulfite, nitrate, bromide, chloride, iodide, Na, K, Ca, **N** **H**_4_^+^, tartarate, citrate, thiosulfate, hydrogen phosphate, and azide. 50-fold amount of Fe^2+^, Cd^2+^, Cr^3+^, Mo^VI^, Ce^IV^, Sb^3+^, Cr^VI^, Se^IV^, Fe^3+^, Ag^+^, Ba^2+^, As^3+^, Pb^2+^, In^3+^, Tl^+^, Mn^2+^, Pd^2+^, V^V^, Be^2+^, Sn^2+^, Bi^2+^, Zn^2+^, Hg^+^, and Hg^2+^. Cobalt, iron, and copper are interfering ions in spectrophotometeric determination of nickel using furildioxime [15]. Here, using nonionic micellar solutions, no interference was observed from iron and copper while cobalt interference remains problem and was selectively removed using modified XAD-16.

Sample solution (5 mL) containing different metal ions including Ni and cobalt was passed through column packed with 0.2 g of modified XAD-16. Nitroso R salt complexes of both Ni and cobalt were retained on column. Therefore, in order to recover retained Ni, column was washed with 2.5 mL of 1 N HNO_3_ [12]. Cobalt-furildioxime complex is stable at lower pH (1 N HNO_3_) thus remain attached with modified resin while Ni-furildioxime decomposes and gets eluted with acid. The pH of eluent was neutralized with NaOH and 50 or 500 *μ*Ls injected in the flowing stream of reagent as mentioned above.

### 3.2. Applications

#### 3.2.1. Determination of Nickel in Synthetic Mixtures

Several synthetic mixtures of varying compositions containing nickel and diverse ions of known concentration were determined by the present method. The results are shown in Table 3.

#### 3.2.2. Determination of Nickel in Cigarette Samples

The proposed method was applied to the determination of nickel tobacco samples. 5 g of each tobacco sample was placed in a breaker of 100 mL capacity and after adding 2 mL of concentrated HNO_3_, the sample was heated almost to dryness on a hot plate at 110°C, then 2 mL of concentrated HNO_3_ was again added to the residue and the solution was heated at 150°C for 2 h. After adding a further 2 mL of concentrated HNO_3_ and 1 mL of 60% HClO_4_, the solution was heated at 150°C for 4 h until white fumes appeared. This procedure was repeated twice. Solution was cooled, filtered, and diluted with deionized water. The nickel content was assayed by standard addition method. Results are summarized in Table 4.

#### 3.2.3. Validation of Method

Some of the reference materials were analysed by proposed method and % recovery was calculated. The results are shown in Table 5.

## 4. Conclusion

A highly selective, sensitive, simple, and rapid method for the determination of nickel using furildioxime in nonionic micellar medium has been developed. Use of micelles eliminates the toxic organic solvents, otherwise, necessary. Using homogenous environment of organized assemblies, the system was coupled with FIA, which increases sample throughput and minimizes sample consumption. The method was successfully applied for the determination of nickel in tobacco samples and complex synthetic mixtures.

## Figures and Tables

**Table 1:** Selected chemical and FIA parameters obtained with optimization experiments.

Parameter	Studied range	Selected value
Size of sample loop (*μ*L)	50–500	500
Reagent flow rate (mL min^−1^)	1.0–4.2	1.2
Length of reaction coil (cm)	50–300	100
pH	3.5–12.0	9.0
Concentration of reagent (%)	0.04–0.25	0.08
Brij-35 (%)	0.01–2.0	0.01
NH_4_Cl/NaOH (M)	0.04–0.4	0.2

**Table 2:** Salient features of the proposed method.

Parameter	Optimized value
pH	9.0
Linear range (*μ*g mL^−1^)	0.02–30
Detection Limit (ng mL^−1^)	10
Dispersion Coefficient	4.0
Reproducibility	0.0–0.2
Sample throughput	120 samples per hour

**Table 3:** Determination of nickel in synthetic mixtures.

Sample	Composition of mixture	Nickel in *μ*g mL^−1^		
		Added	Found	% Recovery
A	Nickel	2.0	2.0	100
B	A + V^V^ (25 fold)	2.0	1.8	90
C	B + Zn^2+^ (25 fold) + Ca (25 fold)	2.0	1.8	90
D	C + Fe (25 fold) + Cr^VI^ (25 fold)	2.0	2.2	110

**Table 4:** Determination of nickel in tobacco samples.

Sample	Amount of nickel *μ*g g^−1^	RSD (%), *n* = 3
Sample-1	0.760	1.3
Sample-2	0.110	2.2
Sample-3	0.75	1.4
Sample-4	0.50	2.4
Sample-5	0.00	0.00
Sample-6	0.125	3.4
Sample-7	0.60	1.4
Sample-8	0.70	2.1
Sample-9	0.150	2.9

**Table 5:** Determination of nickel in Certified Reference Materials.

CRM	Composition of CRM	Nickel found	% recovery
Alloy	50% nickel, 50% aluminium	49.99	99.9

BAS (69b) stainless steel	18.6% Cr, 9.35% nickel	9.32	99.6

BCS 261, stainless steel	0.083% C, 0.39% Si, 17.2% Cr, 13.08% Ni, 0.71% Ta & Nb, 0.66% Mn	19.0	105
